# Neurofilament light chain is increased in the parahippocampal cortex and associates with pathological hallmarks in Parkinson’s disease dementia

**DOI:** 10.1186/s40035-022-00328-8

**Published:** 2023-01-20

**Authors:** Irene Frigerio, Max A. Laansma, Chen-Pei Lin, Emma J. M. Hermans, Maud M. A. Bouwman, John G. J. M. Bol, Yvon Galis-de Graaf, Dagmar H. Hepp, Annemieke J. M. Rozemuller, Frederik Barkhof, Wilma D. J. van de Berg, Laura E. Jonkman

**Affiliations:** 1 Section Clinical Neuroanatomy and Biobanking, Department of Anatomy and Neurosciences, Amsterdam UMC Location Vrije Universiteit Amsterdam, De Boelelaan 1118, Amsterdam, The Netherlands; 2 Amsterdam Neuroscience, Neurodegeneration, Amsterdam, The Netherlands; 3 Amsterdam Neuroscience, Brain Imaging, Amsterdam, The Netherlands; 4 Department of Neurology, Amsterdam UMC Location Vrije Universiteit Amsterdam, De Boelelaan 1117, Amsterdam, The Netherlands; 5 Department of Pathology, Amsterdam UMC Location Vrije Universiteit Amsterdam, De Boelelaan 1117, Amsterdam, The Netherlands; 6 Department of Radiology and Nuclear Medicine, Amsterdam UMC Location Vrije Universiteit Amsterdam, De Boelelaan 1117, Amsterdam, The Netherlands; 7 Institutes of Neurology and Healthcare Engineering, University College London, London, UK

**Keywords:** Neurofilament, NfL, Axonal degeneration, Parkinson’s disease, Parkinson’s disease dementia, Dementia with Lewy bodies, Cortical thickness, Cortical atrophy, Mean diffusivity

## Abstract

**Background:**

Increased neurofilament levels in biofluids are commonly used as a proxy for neurodegeneration in several neurodegenerative disorders. In this study, we aimed to investigate the distribution of neurofilaments in the cerebral cortex of Parkinson’s disease (PD), PD with dementia (PDD) and dementia with Lewy bodies (DLB) donors, and its association with pathology load and MRI measures of atrophy and diffusivity.

**Methods:**

Using a within-subject post-mortem MRI-pathology approach, we included 9 PD, 12 PDD/DLB and 18 age-matched control donors. Cortical thickness and mean diffusivity (MD) metrics were extracted respectively from 3DT1 and DTI at 3T in-situ MRI. After autopsy, pathological hallmarks (pSer129-αSyn, p-tau and amyloid-β load) together with neurofilament light-chain (NfL) and phosphorylated-neurofilament medium- and heavy-chain (p-NfM/H) immunoreactivity were quantified in seven cortical regions, and studied in detail with confocal-laser scanning microscopy. The correlations between MRI and pathological measures were studied using linear mixed models.

**Results:**

Compared to controls, p-NfM/H immunoreactivity was increased in all cortical regions in PD and PDD/DLB, whereas NfL immunoreactivity was increased in the parahippocampal and entorhinal cortex in PDD/DLB. NfL-positive neurons showed degenerative morphological features and axonal fragmentation. The increased p-NfM/H correlated with p-tau load, and NfL correlated with pSer129-αSyn but more strongly with p-tau load in PDD/DLB. Lastly, neurofilament immunoreactivity correlated with cortical thinning in PD and with increased cortical MD in PDD/DLB.

**Conclusions:**

Taken together, increased neurofilament immunoreactivity suggests underlying axonal injury and neurofilament accumulation in morphologically altered neurons with increased pathological burden. Importantly, we demonstrate that such neurofilament markers at least partly explain MRI measures that are associated with the neurodegenerative process.

**Supplementary Information:**

The online version contains supplementary material available at 10.1186/s40035-022-00328-8.

## Background

Parkinson’s disease (PD) is a common neurodegenerative disease with a heterogeneous clinical presentation, which is diagnosed when bradykinesia and tremor and/or rigidity are present [1]. Cognitive impairment is common in PD, leading to Parkinson’s disease dementia (PDD) in up to 80% of PD patients [2–5]. However, when patients develop dementia before or within one year of motor symptom onset, the disease is diagnosed as dementia with Lewy bodies (DLB) [6, 7]. Pathologically, PD, PDD and DLB are defined as synucleinopathies [8], since they are characterized by the accumulation of alpha-synuclein (αSyn) in the form of Lewy bodies (LBs) and Lewy neurites (LNs), which are abundant in the cortex of demented patients [9]. In addition, PDD and DLB patients frequently show Alzheimer’s disease (AD) co-pathology, such as amyloid-beta (Aβ) plaques and phosphorylated-tau (p-tau) neurofibrillary tangles (NFT) [10, 11]. The load of AD co-pathology varies greatly between cases, ranging from cases with no to high AD neuropathological changes [6, 7, 12], but generally DLB patients are likely to show high prevalence of AD neuropathological changes [11]. However, the pathological hallmarks of PDD and DLB largely overlap, and only few neuropathological differences between the two have been described [6, 13, 14].

In addition to pathology load, there is growing evidence for the presence of axonal degeneration in PD, PDD and DLB [15, 16]. Neurofilaments in cerebrospinal fluid (CSF) and blood are commonly used as a proxy of axonal degeneration in many neurodegenerative disorders [15, 16]. Neurofilaments are neuronal-specific proteins which consist of neurofilament light (NfL), medium (NfM) and heavy chains (NfH) [15]. CSF and blood NfL levels have been shown to be elevated in PD, more abundantly in PDD and DLB [17–21], and associated with higher levels of CSF αSyn and p-tau biomarkers [22] as well as with measures of cognitive decline [16–18, 20, 23, 24]. However, the distribution pattern of NfL and phosphorylated-neurofilament medium and heavy chain (p-NfM/H) in cortical brain regions, and to which extent these neurofilaments are related to pathological accumulation in PD, PDD and DLB are yet to be determined.

Atrophy and altered microstructural integrity of the cortex can be captured with MRI outcome measures such as cortical thickness and mean diffusivity (MD), and may be a proxy of underlying neurodegeneration [25]. While PDD and DLB patients show more pronounced cortical atrophy and increased cortical MD compared to cognitively unimpaired elderly [26–29], PD patients show only subtle changes [30–32]. Cortical atrophy and increased cortical MD have been shown to correlate with plasma NfL in several cortical regions in *de-novo* PD [33]. However, little is known about the relation between regional distribution of neurofilaments and MRI-derived microstructural measures in the brains of PD, PDD and DLB.

The aim of the current study was to unravel the regional distribution of cortical neurofilaments, and its association with pathology load and MRI measures of cortical thickness and diffusivity in the brains of clinically-defined and pathologically-confirmed PD, PDD/DLB and control brain donors, using a within-subject post-mortem MRI-pathology approach [34]. Results of this study will increase knowledge on the regional distribution of neurofilaments in the cortex in PD, PDD and DLB, and to what extent this is related to pathological accumulation and reflected by cortical thickness and diffusivity measures, thereby contributing to the understanding of the pathological underpinnings of MRI.

## Materials and methods

### Donor inclusion

In collaboration with the Netherlands Brain Bank (NBB; http://brainbank.nl) we included 21 Lewy Body disease donors who could be further subdivided into 9 PD, 7 PDD and 5 DLB based on clinical presentation [1, 5, 6]. Age at diagnosis (at symptom onset) and disease duration (age at death minus age at diagnosis) were extracted from the clinical files of all donors. Neuropathological diagnosis was confirmed by an expert neuropathologist (A.R.) and performed according to the international guidelines of the Brain Net Europe II (BNE) consortium (https://www.brainbank.nl/about-us/brain-net-europe/) [35–37]. Additionally, 18 age- and sex-matched and pathologically confirmed controls with no records of neurological symptoms at diagnosis, were selected from the Normal Aging Brain Collection Amsterdam (NABCA; http://nabca.eu) [34]. All donors signed an informed consent for brain donation and the use of material and clinical information for research purposes. The procedures for brain tissue collection of NBB and NABCA have been approved by the Medical Ethical Committee of Amsterdam UMC, Vrije Universiteit Amsterdam. For donor characteristics, see Additional file 1: Table S1.

### Post-mortem in-situ MRI acquisition

**Fig. 1:**
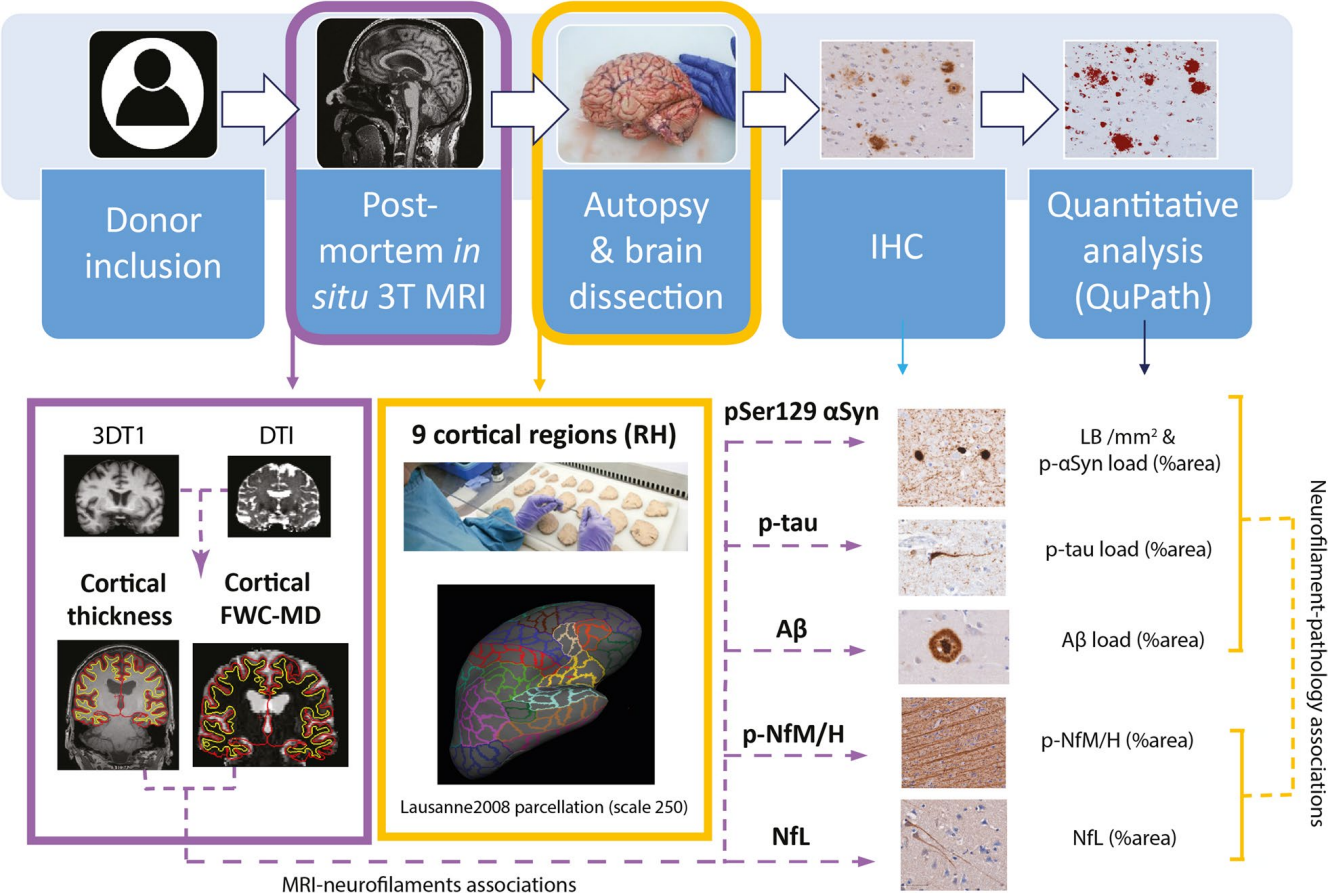
Workflow. After donor inclusion, post-mortem in-situ 3DT1 and DTI were collected [34], from which cortical thickness was derived with Freesurfer [41] and free-water corrected cortical mean diffusivity (FWC-MD) was derived with FSL [44], DIPY [47, 48] and Freesurfer [41] (purple box). After the MRI scan, autopsy was performed, and 9 cortical regions were selected from the right hemisphere (RH) for further analysis. To match these regions to the MR images, regions of interest were selected from the Lausanne atlas [42, 43] (yellow box). Brain tissue was processed for immunohistochemistry against phosphorylated Ser129 α-synuclein (pSer129-αSyn, clone EP1536Y), phosphorylated-tau (p-tau, clone AT8), amyloid β (Aβ, clone 4G8), neurofilament light chain (NfL, amino acid sequence 1-376) and phosphorylated neurofilaments medium and heavy chains (p-NfM/H, clone SMI312), which were quantified using QuPath [50]. The correlations between neurofilament immunoreactivity and pathology load, and between neurofilament immunoreactivity and MRI outcome measures were investigated via linear mixed models (yellow and purple dashed arrows, respectively). Aβ: amyloid beta; FWC-MD: free water corrected mean diffusivity; IHC: immunohistochemistry; LB: Lewy Body; NfL: neurofilament light chain; pSer129-αSyn: phosphorylated Ser129 alpha synuclein; p-NfM/H: phosphorylated neurofilament medium and heavy chain; p-tau: phosphorylated tau; RH: right hemisphere

Post-mortem in-situ (brain in cranium) MRI scans were acquired according to a previously described pipeline [34] (Fig. 1), with a post-mortem delay (interval between death and MRI) of maximum 13 h for all brain donors. Briefly, scans were acquired on a 3T scanner (Signa-MR750, General Electric Medical Systems, Milwaukee, WI) with an eight-channel phased-array head-coil. The following pulse-sequences were performed for all subjects: (i) sagittal 3D T1-weighted fast spoiled gradient echo (repetition time [TR] = 7 ms, echo time [TE] = 3 ms, flip angle = 15°, 1-mm-thick axial slices, in-plane resolution = 1.0 × 1.0 mm^2^); (ii) sagittal 3D fluid attenuation inversion recovery (FLAIR; TR = 8000 ms, TE = 130 ms, inversion time [TI] = 2000–2500 ms, 1.2-mm-thick axial slices, in-plane resolution = 1.11 × 1.11 mm^2^), with TI corrected for post-mortem delay; (iii) diffusion-weighted imaging (DWI) axial 2D echo-planar imaging with diffusion gradients applied in 30 non-collinear directions, TR/TE = 7400/92 ms, slices thickness of 2.0 mm, in-plane resolution = 2.0 × 2.0 mm^2^, b = 1000 s/mm^2^, and 5 b0 scans. To allow for offline distortion correction of the images, b0 images with reversed phase-encoding direction were acquired using the same sequence parameters.

### MRI analysis

#### Cortical thickness and brain volume assessment

To minimize the impact of age-related white matter abnormalities (e.g. vascular change) on automated segmentations, the 3D-T1 images were lesion-filled [38] as previously described [39, 40]. Image processing was performed using Freesurfer, version 6.0 (http://surfer.nmr.mgh.harvard.edu) [41]. For each subject, nine cortical regions of interest (ROIs) in the right hemisphere were selected from the Lausanne atlas parcellation [42, 43], to closely match the location of the cortical regions dissected at autopsy. Details on the Lausanne atlas labels corresponding to the ROIs in the present study can be found in Additional file 1: Table S2, and included the superior frontal gyrus, anterior and posterior cingulate gyrus, anterior insula, middle temporal gyrus, superior parietal gyrus, entorhinal cortex, parahippocampal and fusiform gyrus. Cortical thickness was measured as the perpendicular distance from the grey/white matter boundary to the corresponding pial surface. Quality of parcellations was assessed using the ENIGMA guidelines for quality control (see http://enigma.ini.usc.edu/protocols/imaging-protocols/). Furthermore, post-mortem normalized brain volume, and normalized grey and white matter volumes were obtained from the T1-weighted images using Structural Image Evaluation, using Normalisation, of Atrophy (SIENAX) (part of FSL 5.0.9; http://fsl.fmrib.ox.ac.uk/), which estimates brain tissue volume normalized for skull size [44].

#### Diffusion tensor imaging (DTI) pre-processing and free water correction

DTI and reference scans of opposite phase encodings (acquired along anterior–posterior and posterior-anterior directions) were collected for all 9 PD donors, 11 out of 12 PDD/DLB donors, and 14 out of 18 control donors (see Additional file 1: Table S1). DTI was first corrected for susceptibility distribution and eddy current-induced geometric distortion using FSL (Eddy and topup) [44] and fitted for single tensors diffusion maps, deriving MD [45], which is a good (bio)marker for grey matter microstructure, where diffusion does not conform to a specific direction [46]. To avoid underestimation by partial volume effects of the CSF, a bi-tensor model for free water correction was performed using an open-resource, python-based script of DIPY, which has been shown to be plausible for using single shell DWI acquisitions to derive free-water corrected MD (FWC-MD) maps [47, 48]. Using Freesurfer version 7.1.1 [41], we performed a within-subject registration of T1-weighted to FWC-MD images. With this registration, the cortical thickness of ROIs was registered to the FWC-MD map and corrected for overestimation into the white matter, by applying a threshold at 50% probability and a limit to the cortical ribbon mask, after which regional cortical FWC-MD values were obtained. Quality of parcellations was manually assessed (I.F.) and, when necessary, poorly parcellated regions were manually corrected or excluded from statistical analysis (i.e. entorhinal cortex of 1 PD case and 3 PDD/DLB cases was excluded). For clarity, FWC-MD is referred to as MD in the manuscript.

#### Tissue sampling

Subsequent to MRI acquisition, brain tissue was collected at autopsy, resulting in a total post-mortem delay (interval between death and autopsy) of maximum 13 h for all brain donors. Formalin-fixed paraffin-embedded tissue blocks (4% formalin, four weeks of fixation) from the following seven regions within the right hemisphere were obtained and processed for immunohistochemistry (IHC): superior frontal gyrus, anterior and posterior cingulate gyri, anterior (dysgranular) insula, middle temporal gyrus, superior parietal gyrus, and hippocampus (including the entorhinal cortex, and parahippocampal and fusiform gyri, as described before) [39, 49].

#### IHC for quantification

Sections (6-µm thick) from tissue blocks of the above-mentioned regions were cut and mounted onto superfrost + glass slides (Thermo Scientific, Waltham, MA). All sections were stained for pSer129-αSyn (clone EP1536Y), Aβ (clone 4G8) and p-tau (clone AT8). Additionally, sections from 7 out of the 9 cortical brain regions (showing significantly higher LB count across groups, i.e., all regions except the posterior cingulate cortex and the superior parietal gyrus) were stained for NfL (amino acid sequence 1–376) and p-NfM-H (clone SMI312) (for information of primary antibodies see Additional file 1: Table S3). Briefly, the sections were deparaffinised, immersed in buffer, and heated to 100 °C in a steam cooker for 30 min for antigen retrieval. The sections were blocked for endogenous peroxidase using 1% hydrogen peroxide and in Tris-buffered saline (TBS; pH 7.4), and consequently in 3% normal donkey serum in TBS (Triton 0.5%). Primary antibodies were diluted in 1% normal donkey serum in TBS (Triton 0.1%) and incubated overnight at 4 °C. Primary antibodies were detected using EnVision (Dako, Glostrup, Denmark), and visualized using 3.3’-Diaminobenzidine (DAB, Dako) with Imidazole (50 mg DAB, 350 mg Imidazole and 30 μl of H_2_O_2_ per 100 ml of Tris–HCl 30 mM, pH 7.6). Between steps, TBS was used to wash the sections. After counterstaining with haematoxylin, the sections were dehydrated and mounted with Entellan (Merck, Darmstadt, Germany).

### Pathological analysis

#### Pathology image processing

Using a whole-slide scanner (Vectra Polaris, 20 × objective), images of immunostained sections were taken and quantified using QuPath 0.2.3 (https://qupath.readthedocs.io/en/0.2/) [50]. ROIs containing all cortical layers were delineated in straight areas of the cortex to avoid over- or underestimation of pathology in sulci and gyri, respectively [51]. Hippocampal sections were segmented according to the method described by Adler et al. [49], where the entorhinal cortex, parahippocampal gyrus and fusiform gyrus were delineated to match the MRI-derived ROIs. Briefly, the entorhinal cortex was delineated from the end of the parasubiculum until layer IV started to be visible [52]; the parahippocampal gyrus started from this point and ended at the collateral sulcus; the fusiform gyrus started at this point and ended at the inferior temporal sulcus (Additional file 2: Fig. S1). For the pSer129-αSyn, NfL and p-NfM/H stainings, the cortex was further segmented into superficial (layers I-III) and deep (layers IV-VI) cortical layers based on the haematoxylin counterstaining. For Aβ and p-tau, the same was done in the entorhinal cortex, parahippocampal and fusiform gyrus, as these are among the first regions to be affected by AD co-pathology [53, 54]. Note that the entorhinal cortex was subdivided into layers I-III (superficial) and lamina dissecans plus layers V-VI (deep) [52]. DAB immunoreactivity was quantified with *in-house* QuPath scripts, using pixel and object classifiers. The outcome measures for pSer129-αSyn were both LB count per mm^2^ (LB/mm^2^) and pSer129-αSyn %area load excluding LBs (for additional information, see Additional file 3: Supplementary Material), while outcome measures for Aβ and p-tau were %area load (Additional file 2: Fig. S2). The outcome measures for neurofilaments were %area load, expressed in the text as %immunoreactivity (Additional file 2: Fig. S3).

#### Multi-label immunofluorescence and confocal microscopy for morphological characterisation of neurofilaments

To study the morphological features of neurofilaments, we performed a multi-label immunofluorescence staining in combination with 3D confocal laser scanning microscopy (CSLM) of p-NfM/H and NfL on a representative case of each group. This was done on the parahippocampal gyrus, as this was the region showing the largest neurofilament alterations between groups. Briefly, sections were deparaffinised, immersed in Tris EDTA pH 9.0, and heated to 100 °C in a steam cooker for 30 min for antigen retrieval. The sections were blocked for endogenous peroxidase using 1% hydrogen peroxide in TBS, and consequently in 3% normal donkey serum in TBS (Triton 0.5%; pH 7.4). Primary antibodies were diluted in 1% normal donkey serum in TBS (Triton 0.1%; pH 7.4) and incubated overnight at 4 °C. Primary antibodies were detected and visualized with donkey anti-mouse Alexa 488 (ThermoFisher, Pittsburgh, PA) and donkey anti-rabbit Alexa 594 (ThermoFisher) targeting p-NfM/H and NfL, respectively. For all stainings, TBS was used to wash the sections between steps. After counterstaining with DAPI (Sigma-Aldrich, St. Louis, MO), the sections were mounted with Mowiol (Sigma-Aldrich) plus anti-fading agent DABCO. Confocal imaging was performed with a Leica TCS SP8 (Leica Microsystems, Germany) using a HC PL PAO CS2 60 × oil objective lens, NA 1.40 and a zoom factor of 2.0. Sections were sequentially scanned for each fluorochrome with a pulsed white light laser at different wavelengths (excitation wavelengths: DAPI at 405 nm; Alexa 488 at 499 nm; Alexa 594 at 598 nm). All signals were detected using gated hybrid detectors in counting mode. Z-stacks (Z = 6 μm; 1024 × 1024 pixels) were taken in the parahippocampal gyrus of a representative control, PD and DLB. After scanning, the images were deconvoluted using CMLE algorithms in Huygens Professional (Scientific Volume imaging; Huygens, The Netherlands; https://svi.nl/Huygens-Professional), and their maximum projections (ImageJ Fiji, National Institute of Health, USA; https://imagej.nih.gov/ij/) or 3D surface rendering reconstructions (Imaris, Oxford instruments 2022: https://imaris.oxinst.com/) were used to represent graphically the structures of interests and their morphologies. In some cases, signal brightness was increased in ImageJ for clarity. Figures were created using Adobe Illustrator (CS6, Adobe Systems incorporated).

### Statistics

Statistical analyses were performed in SPSS 26.0 (Chicago, IL). Normality was tested, and demographics of PD, PDD/DLB and control groups were compared using parametric or non-parametric tests for continuous data, and Fisher exact test for categorical data. The associations between neurofilament immunoreactivity and pathological staging were calculated with Spearman’s correlation. To account for multiple (i.e., 9) brain regions within cases (i.e., nested data), the group differences across all regions and the associations between variables were assessed with multiple linear mixed models. In the case of pathological group differences, the pathological variable of interest was the dependent variable, and age and gender the main effects (i.e., covariates). In the case of MRI group differences, cortical thickness or MD was the dependent variable, and age, sex and post-mortem delay the main effects (i.e., covariates). In the case of global associations between neurofilament immunoreactivity and pathology load (for example NfL and p-tau), the neurofilament immunoreactivity was the dependent variable and the pathological load was the main effect, together with age and gender (i.e., covariates). And lastly, in the case of global associations between MRI measures and pathological markers (for example cortical thickness and NfL), cortical thickness or MD was the dependent variable and neurofilament immunoreactivity or pathology load was the main effect, together with age, gender and post-mortem delay (i.e. covariates). The intercept was included in all analyses as random effect. Post-mortem delay was added as a covariate in MRI-related analyses since it might influence post-mortem MRI-derived biomarkers [55, 56]. For linear mixed model associations, cortical thickness metrics were transformed into z-scores for each brain region as different cortical regions inherently have different thicknesses, which may drive the association. In all analyses, multiple comparisons between groups were corrected with *post-hoc* Bonferroni, and group comparisons were expressed as percentage differences, as in the formula: %difference = [(absolute difference)/mean control]*100. Statistics at the brain region level were corrected for multiple comparisons using the false discovery rate approach (FDR), and FDR-corrected *P*-values are expressed as *P*-FDR [57]. After Bonferroni and FDR correction, *P*-values less than 0.05 were considered significant. Correlation coefficients (*r*) of linear mixed model associations were calculated as in the formula: *r* = (estimate fixed effect * standard deviation-fixed effect) / standard deviation-dependent variable.

## Results

### Cohort description

Demographic, clinical, radiological, and pathological data of PD, PDD/DLB and non-neurological control donors are summarized in Table 1 (and per donor in Additional file 1: Table S1). Sex, age at diagnosis, age at death, and post-mortem delay did not differ between groups, whereas disease duration was shorter in PDD/DLB compared to PD donors (*P* = 0.006), due to the inclusion of DLB donors with shorter disease duration (*n* = 5, mean ± SD, 5 ± 1.6 years). On MRI, normalized brain volume (*P* = 0.011) and normalized grey matter volume (*P* = 0.019), but not normalized white matter volume (*P* = 0.095) were lower in PDD/DLB cases compared to controls. In terms of pathology load, the PDD/DLB group showed more abundant LB count, p-tau and Aβ load compared to controls (number of LB/mm^2^: *P* < 0.001*;* p-tau: *P* = 0.015*;* Aβ: *P* = 0.018) and PD donors (number of LB/mm^2^*: P* = 0.033*;* p-tau: *P* = 0.035; Aβ: *P* = 0.033). Moreover, LBs were more abundant in deep than superficial cortical layers (PD: *P* = 0.004*; PDD/DLB: P* = 0.035), while p-tau and Aβ were more abundant in superficial than deep cortical layers in PDD/DLB (p-tau: *P* = 0.046*;* Aβ: *P* < 0.001). The p-tau load strongly correlated with both LB count (*r* = 0.68, *P* < 0.001) and pSer129-αSyn load (*r* = 0.60, *P* < 0.001) in the PDD/DLB group. More details on regional and layer-specific distribution of pSer129-αSyn, Aβ, and p-tau load, are shown in Additional file 2: Fig. S4. For more details on correlations between pathological hallmarks, see Additional file 1: Table S4.

**Table 1:** Donor’s characteristics

	Control	PD	PDD/DLB
*N*	18	9	12 (7 PDD, 5 DLB)
Sex M/F (%M)	8/10 (44%)	6/3 (67%)	7/5 (58%)
Age at diagnosis (years), mean ± SD	–	62 ± 7	68 ± 12
Disease duration (years), mean ± SD	–	18 ± 5	11 ± 6^ϮϮ^
Age at death (years), mean ± SD	73 ± 8	80 ± 9	78 ± 9
Post-mortem delay (minutes), mean ± SD	524 ± 110	469 ± 153	443 ± 107
Radiologic characteristics			
NBV (L) mean ± SD	1.46 ± 0.07	1.43 ± 0.08	1.38 ± 0.07*
NGMV (L) mean ± SD	0.74 ± 0.05	0.72 ± 0.04	0.70 ± 0.04*
NWMV (L) mean ± SD	0.72 ± 0.03	0.72 ± 0.05	0.68 ± 0.04
Pathological characteristics			
Braak LB stage [55] *N*	18	9***	12***
0/1/2/3/4/5/6	15/2/1/0/0/0/0	0/0/0/0/1/1/7	0/0/0/0/0/0/12
ABC score [56] *N*	18	9	12
A 0/1/2/3	4/13/1/0	1/6/2/0	0/3/5/4**
B 0/1/2/3	3/15/0/0	0/7/2/0	0/5/7/0**
C 0/1/2/3	18/0/0/0	7/2/0/0	5/1/6/0**
Thal phase [52] *N*	18	9	12**
0/1/2/3/4/5	4/8/5/1/0/0	1/3/3/2/0/0	0/2/1/5/3/1
Braak NFT stage [51] *N*	18	9	12***
0/1/2/3/4/5/6	3/11/4/0/0/0/0	0/2/5/1/1/0/0	0/0/5/2/5/0/0
CAA type [57] *N*	18	9	12
No CAA/type 1/ type 2	14/1/3	9/0/0	5/4/3

Data are mean ± standard deviation (SD). CAA: cerebral amyloid angiopathy; DLB: dementia with Lewy bodies; F: females; LB: Lewy body; M: males; NBV: normalized brain volume; NFT: neurofibrillary tangles; NGMV: normalized grey matter volume; NWMV: normalized white matter volume; PD: Parkinson’s disease; PDD: Parkinson’s disease dementia; SD: standard deviation. *P < 0.05, **P < 0.01, ***P < 0.001 when compared to controls, and ^ϮϮ^*P* < 0.01 when compared to the PD group

### Increase in neurofilament immunoreactivity in the cortex of PD and PDD/DLB

**Fig. 2:**
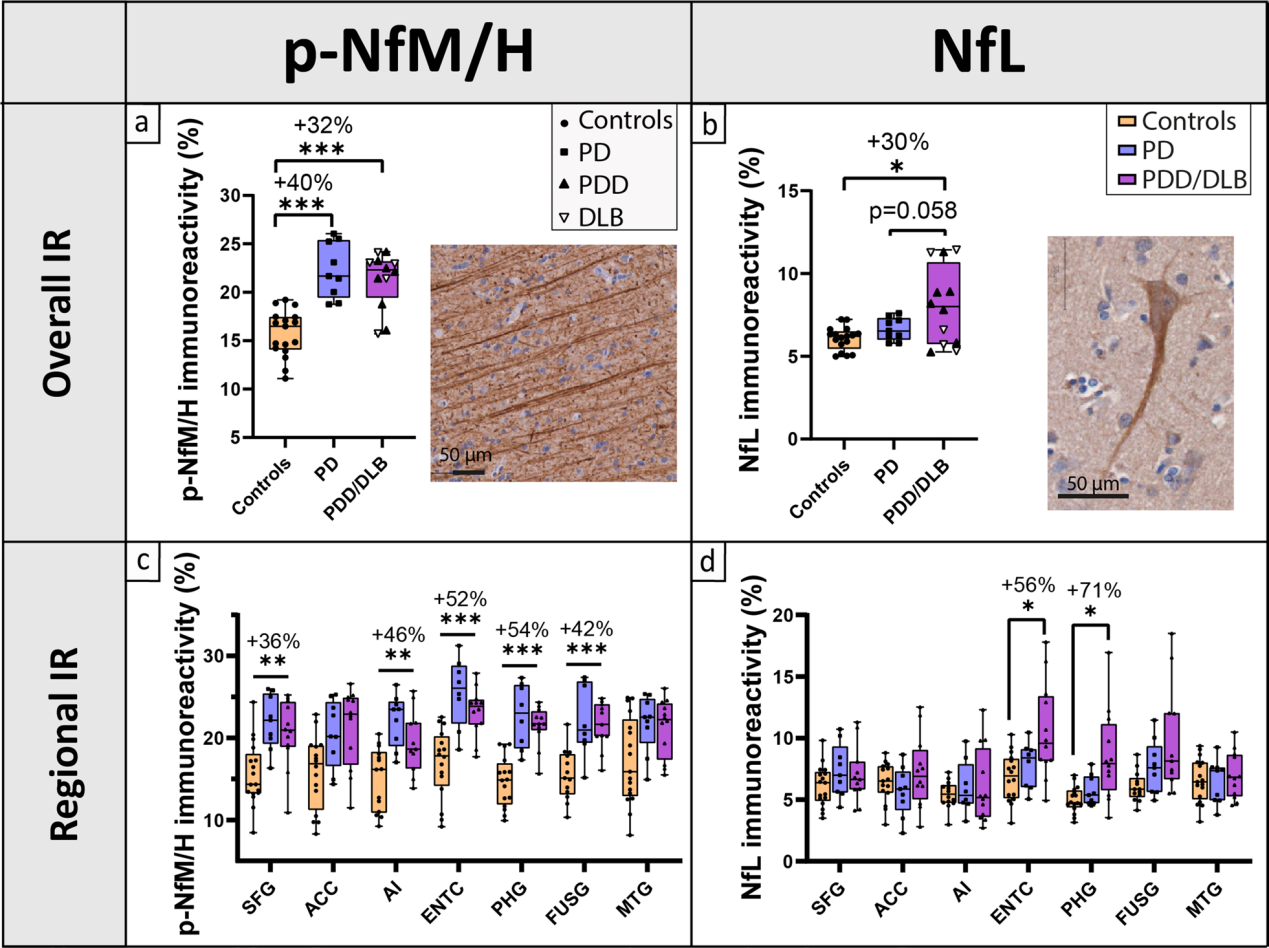
Cortical neurofilament immunoreactivity (IR) and distribution across cortical brain regions. (**a**, **c**) Cortical p-NfM/H and (**b**, **d**) NfL immunoreactivity are shown for controls, PD and PDD/DLB groups. The first row (**a**, **b**) shows the overall neurofilament immunoreactivity across all cortical regions examined, with percentage differences compared to controls indicated at the top of the graph; every data point represents the averaged neurofilament immunoreactivity across all cortical regions of a donor , and the clinical groups are indicated by shapes, showing that PDD and DLB donors behaved similarly. Moreover, images of p-NfM/H (**a**) and NfL stainings (**b**) show that p-NfM/H targets the axons, while NfL targets both the neuronal soma and its processes. The second row (**c** and **d)** shows the regional neurofilament immunoreactivity in the 7 cortical regions analysed. Percentage differences (**c**) between p-NfM/H immunoreactivity in PD vs controls and (**d**) between NfL immunoreactivity in PDD/DLB vs controls are shown for brain areas hosting a significant difference. From these data, we can infer that p-NfM/H was uniformly increased across the cortical areas analysed in both PD and PDD/DLB, while NfL was increased specifically in the entorhinal and parahippocampal cortex in PDD/DLB. *P < 0.05, **P < 0.01, ***P < 0.001 when compared to controls. ACC: anterior cingulate cortex; AI: anterior insula; DLB: Dementia with Lewy Bodies; ENTC: entorhinal cortex; FUSG: fusiform gyrus; IR: immunoreactivity; MTG: middle temporal gyrus; NfL: neurofilament light chain; PHG: parahippocampal gyrus; PD: Parkinson’s disease; PDD: Parkinson’s disease dementia; p-NfM/H: phosphorylated neurofilament medium and heavy chain; SFG: superior frontal gyrus

P-NfM/H and NfL immunoreactivity were investigated in seven cortical areas. P-NfM/H staining was observed in axons, while NfL staining was seen in the neuronal soma and its processes (Fig. 2a, b). Both p-NfM/H and NfL immunoreactivity positively correlated with age (p-NfM/H: *r* = 0.33, *R*^2^ = 11%, *P* = 0.011; NfL in superficial cortical layers: *r* = 0.23, *R*^2^ = 5%, *P* = 0.020), and p-NfM/H was significantly higher in males compared to females (*P* = 0.007) (Additional file 2: Fig. S5). P-NfM/H immunoreactivity was higher in deep compared to superficial cortical layers in all groups (controls: + 38% compared to superficial layers, *P* < 0.001; PD: + 22%, *P* < 0.001; PDD/DLB: + 23%, *P* < 0.001), while NfL immunoreactivity showed the opposite, being more abundant in superficial than in deep cortical layers in all groups (controls: + 26% compared to deep layers, *P* < 0.001; PD: + 27%, *P* < 0.001; PDD/DLB: + 21%, *P* = 0.003). For more details on layer-specific distribution of neurofilaments, see Additional file 2: Fig. S6.

Overall, p-NfM/H immunoreactivity was significantly increased in both PD (*P* < 0.001) and PDD/DLB (*P* < 0.001) compared to controls by 40% and 32% respectively, and did not differ between PD and PDD/DLB (*P* = 1.000) (Fig. 2a, and Additional file 2: Fig. S3a for representative images). To investigate whether the increase in p-NfM/H immunoreactivity in the PDD/DLB group was driven by a specific clinical phenotype (PDD or DLB), we explored the difference in p-NfM/H immunoreactivity between these groups, but observed no differences. Regionally, p-NfM/H immunoreactivity was significantly increased in both PD and PDD/DLB groups compared to controls in the superior frontal gyrus (PD: + 36%, *P*-FDR = 0.009; PDD/DLB: + 27%, *P*-FDR = 0.036), entorhinal cortex (PD: + 52%, *P*-FDR = 0.001; PDD/DLB: + 38%, *P*-FDR = 0.003), parahippocampal gyrus (PD: + 54%, *P*-FDR < 0.001; PDD/DLB: + 46%, *P*-FDR < 0.001), fusiform gyrus (PD: + 42%, *P*-FDR = 0.019; PDD/DLB: + 39%, *P*-FDR = 0.005) and anterior insula (PD: + 46%, *P*-FDR = 0.003; PDD/DLB: + 29%, *P*-FDR = 0.049) (Fig. 2c).

Overall, NfL immunoreactivity was significantly increased in the PDD/DLB group compared to controls (+ 30%, *P* = 0.037), and tended to be increased in PDD/DLB compared to PD (*P* = 0.058), while it did not differ in PD compared to controls (*P* = 1.000) (Fig. 2b, and Additional file 2: Fig. S3b for representative images). To investigate whether the increase in NfL immunoreactivity in the PDD/DLB group was driven by a specific clinical phenotype (PDD or DLB), we explored the difference in NfL immunoreactivity between these groups, but observed no differences. Regionally, NfL immunoreactivity was increased specifically in the entorhinal cortex (+ 56%, *P*-FDR = 0.028) and the parahippocampal gyrus (+ 71%, *P*-FDR = 0.016) in PDD/DLB compared to controls (Fig. 2d).

Taken together, p-NfM/H immunoreactivity was uniformly increased across all cortical areas analysed in all patient groups, while increased NfL immunoreactivity was observed specifically in the entorhinal cortex and the parahippocampal gyrus in PDD/DLB.

### Accumulation and fragmentation of neurofilaments in diseased neurons in PDD/DLB

To better understand the observed increase in p-NfM/H and NfL immunoreactivity in PD and PDD/DLB, we examined the morphological differences of both neurofilaments in the region that was most affected, the parahippocampal gyrus, using multi-label immunofluorescence and 3D CSLM.

**Fig. 3:**
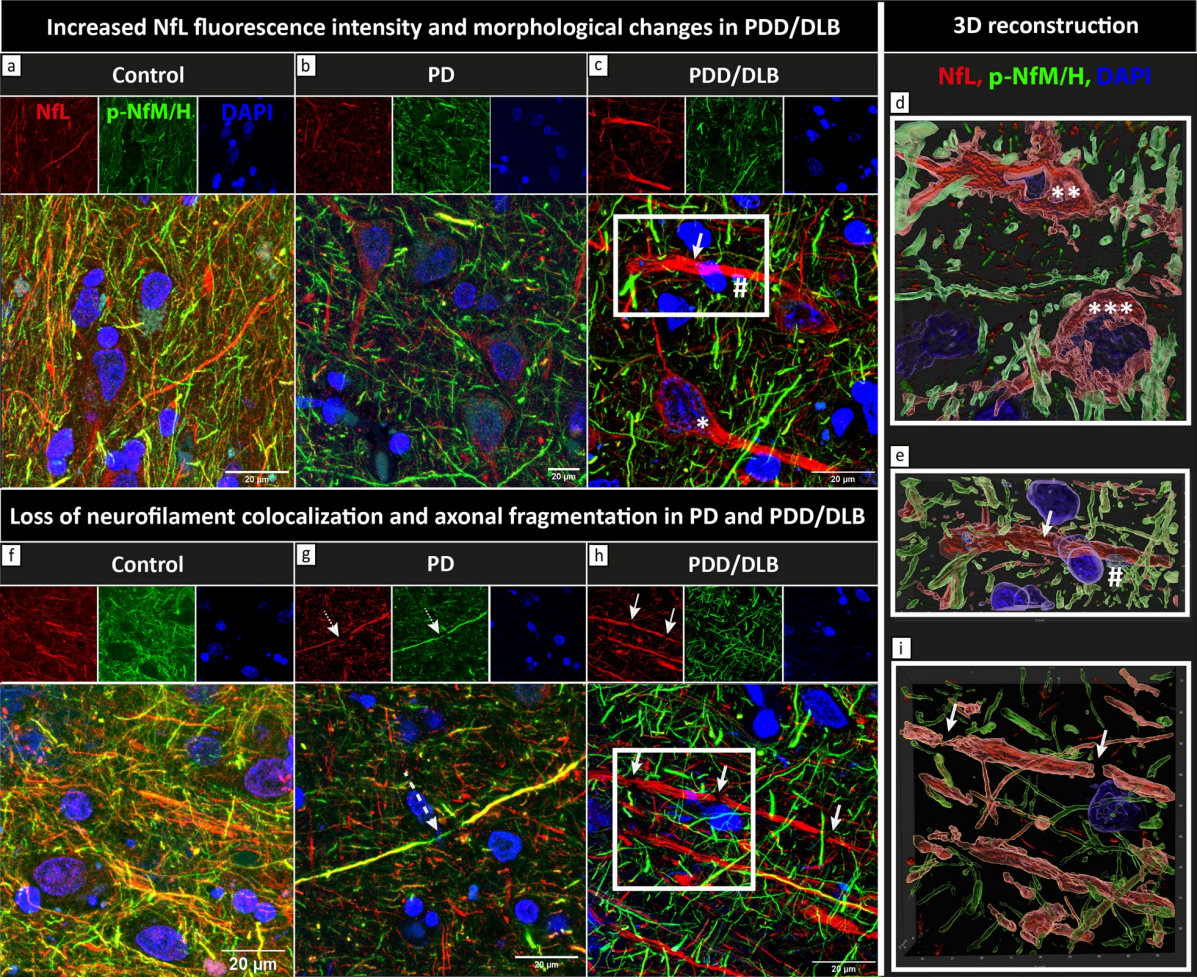
NfL accumulation and fragmentation, and loss of neurofilament colocalization in PD and PDD/DLB. Maximum projections (Z-stacks, *Z* = 6 μm) of fluorescence stainings of NfL (red) and p-NfM/H (green) together with DAPI (blue) in the parahippocampal gyrus of a representative control (*left,* control number 10 in Additional file 1: Table S1), PD (*middle,* PD number 3 in Additional file 1: Table S1) and PDD/DLB (*right,* here a DLB donor, PDD/DLB number 12 in Additional file 1: Table S1) are shown. **a**–**c** show the difference in NfL staining pattern in control, PD and PDD/DLB, respectively. NfL markedly accumulated in neuronal somas and proximal axons in PDD/DLB cases, reflecting the higher NfL immunoreactivity described quantitatively in Fig. 2d. High NfL immunoreactivity was found in neuronal somas showing neurodegenerative morphological features, such as (**c**) neurons with nuclear fragmentation and swelling (*), and (**d**) corkscrew (**) and ballooned (***) appearances (panel **d** shows a zoom-in 3D surface-reconstruction of neurodegenerative neurons). Moreover, (**c**) NfL accumulation was also identified in a proximal axon showing fragmentation (arrow), closely tighten by a glial cell (#); **e** shows the zoom-in 3D surface-reconstruction of the white square in **c**, showing axonal fragmented morphology (arrow) and a glial cell wrapping the degenerating axon (#). **f**–**h** show the colocalization (yellow) of NfL and p-NfM/H in control, PD and PDD/DLB, respectively, which was reduced in PD and PDD/DLB. Moreover, accumulation and fragmentation of NfL staining patterns were observed in several axons, where (**g**) NfL seemed to fragment within a p-NfM/H axon in PD (dashed arrow) and (**h)** axonal swellings intermitted axonal fragmentations in PDD/DLB (arrows). **i** Zoom-in 3D surface-reconstruction of the white square in **h**, clearly showing fragmentation of the NfL-positive axon (arrows). DLB: Dementia with Lewy bodies; NfL: neurofilament light chain; PD: Parkinson’s disease; PDD: Parkinson’s disease dementia; p-NfM/H: phosphorylated neurofilament medium and heavy chain

First, NfL was much more present in neuronal somas and apical axons of PDD/DLB compared to PD and controls (Fig. 3a–c). Specifically, NfL seemed to accumulate in neurons showing neurodegenerative morphologies, such as nuclear fragmentation and swelling (Fig. 3c), and ballooning and corkscrew deformation of the soma (Fig. 3d). Second, accumulation and fragmentation of NfL staining patterns were observed in several axons, where axonal swellings intermitted axonal fragmentations in PD and, more abundantly, in PDD/DLB (Fig. 3e, g, h, and i), which were not observed in controls. Third, we also observed glial cells close to an apparently fragmented axon in PDD/DLB (Fig. 3c, e). Lastly, while p-NfM/H and NfL co-localized in most of the control axons, axons in the deep cortical layers of PD and PDD/DLB cases were mostly positive for p-NfM/H or NfL, rarely showing co-localization of the two neurofilaments (Fig. 3f–h). This was also supported by our quantitative data, showing an absence of significant correlation between the two neurofilaments in PD and PDD/DLB groups, which was present in controls (Additional file 2: Fig. S7).

Taken together, NfL seemed to accumulate in diseased neurons in PDD/DLB and to fragment axons in PD and, more abundantly, in PDD/DLB. Moreover, both neurofilaments showed less overlap in immunoreactivity profile in PD and PDD/DLB groups.

### Cortical neurofilament immunoreactivity correlates with pathological staging

To better understand the relationship between the observed increases in p-NfM/H and NfL immunoreactivity in the parahippocampal gyrus (the most affected region) and pathological disease staging, we tested the correlation of neurofilament immunoreactivity with Braak LB stage, Braak NFT stage and Thal phase.

P-NfM/H immunoreactivity in the parahippocampal gyrus positively and strongly correlated with Braak LB stage (*r*_s_ = 0.75, *P* < 0.001) and Braak NFT stage (*r*_s_ = 0.59, *P* < 0.001), and moderately with Thal phase (*r*_s_ = 0.44, *P* = 0.007) in the whole cohort (Additional file 2: Fig. S8). Similarly, NfL immunoreactivity in the parahippocampal gyrus positively and moderately correlated with Braak LB stage (*r*_s_ = 0.44, *P* = 0.007), and strongly with Braak NFT stage (*r*_s_ = 0.55, *P* < 0.001) and Thal phase (*r*_s_ = 0.54, *P* = 0.001) (Additional file 2: Fig. S8).

### Cortical neurofilament immunoreactivity correlates with pSer129-αSyn load, and more strongly with p-tau load in PDD/DLB

**Fig. 4:**
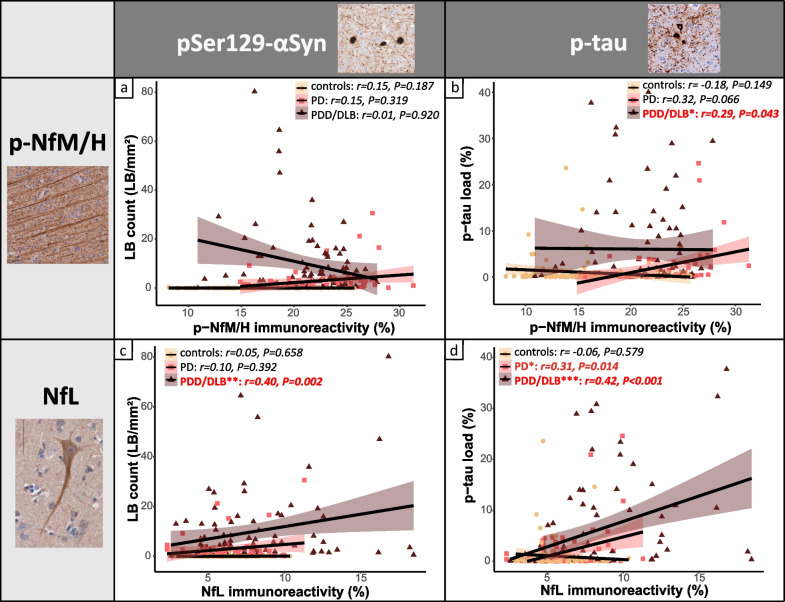
Cortical neurofilament immunoreactivity correlates with pSer129-αSyn and p-tau load. Correlation of (**a** and **b**) cortical p-NfM/H and (**c** and **d**) NfL immunoreactivity with pSer129-αSyn-positive LB count and p-tau load, respectively**.** Every data point represents a cortical region of a donor, and the groups are indicated by different colours. For each group, the regression line is shown, together with its colour-coded standard error (orange for controls, pink for PD and brown for PDD/DLB). Significant correlations are highlighted in bold red in the upper box in every panel. Legend: correlation significant at *P < 0.05, **P < 0.01, ***P < 0.001. DLB: Dementia with Lewy bodies; LB: Lewy Body; NfL: neurofilament light chain; PD: Parkinson’s disease; PDD: Parkinson’s disease dementia; p-NfM/H: phosphorylated neurofilament medium and heavy chain; p-tau: phosphorylated tau

No correlations were found between p-NfM/H immunoreactivity and pSer129-αSyn pathology in any group across all regions (*P* > 0.05, Fig. 4a). On the other hand, p-NfM/H positively correlated with p-tau load in the PDD/DLB group across all regions (*r* = 0.29, *R*^2^ = 8%, *P* = 0.043), which was a trend in PD (*r* = 0.32, *P* = 0.066), and absent in controls (*P* = 0.149) (Fig. 4b). No correlation was found with Aβ load (*P* > 0.05). The associations were not specific to the superficial or deep cortical layers (for all correlations, see Additional file 1: Table S5).

NfL positively correlated with pSer129-αSyn pathology in PDD/DLB across all regions (LB count: *r* = 0.40, *R*^2^ = 16%, *P* = 0.002; pSer129-αSyn load: *r* = 0.36, *R*^2^ = 13%, *P* = 0.005), but not in the PD group (LB count: *P* = 0.392; pSer129-αSyn load: *P* = 0.910) (Fig. 4c). Moreover, NfL also positively correlated with p-tau load in PDD/DLB (*r* = 0.42, *R*^2^ = 18%, *P* < 0.001) and PD (*r* = 0.31, *R*^2^ = 10%, *P* = 0.014), but not in controls across all regions (*P* = 0.579) (Fig. 4d). When both pSer129-αSyn and p-tau load were considered, NfL correlated with p-tau load (*r* = 0.24, *R*^2^ = 6%, *P* = 0.030) but not pSer129-αSyn load (*P* = 0.195). Taken together, it seems that NfL correlated more strongly with p-tau than with pSer129-αSyn load. No correlation was found between NfL and Aβ load (*P* > 0.05). Additionally, the associations were not specific to superficial or deep cortical layers (for all correlations, see Additional file 1: Table S6).

Taken together, in PDD/DLB, higher p-NfM/H immunoreactivity correlated with higher p-tau load, while NfL immunoreactivity correlated with higher pSer129-αSyn load, but more strongly with higher p-tau load. None of the neurofilaments correlated with Aβ load.

### Cortical neurofilament immunoreactivity is reflected by MRI cortical thickness in PD

To investigate whether MRI-derived cortical thickness is sensitive to regional neurofilament changes in the cortex of PD and PDD/DLB donors, we assessed whether regional neurofilament immunoreactivity correlated with cortical thickness across the included brain regions.

On MRI, neither PD (*P* = 1.000) nor PDD/DLB cases (*P* = 0.756) showed cortical thickness differences compared to controls across and within all cortical brain regions (all *P*-FDR > 0.05) (Additional file 1: Fig. S9a and b). Moreover, cortical thickness did not correlate with any pathological hallmark (*P* > 0.05) (Additional file 2: Fig. S10a and c, and Additional file 1: Table S7).

**Fig. 5:**
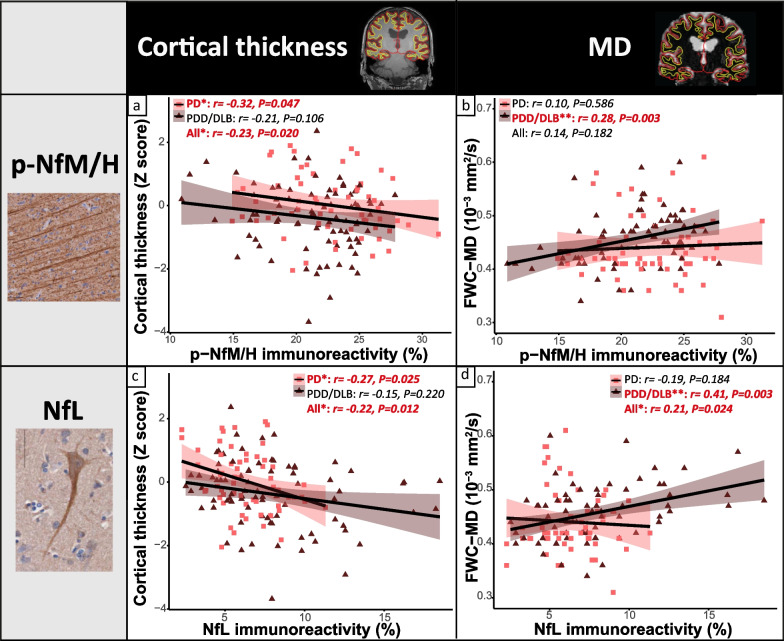
Neurofilament immunoreactivity correlates with decreased cortical thickness in PD and increased cortical MD in PDD/DLB. (**a**, **b**) Correlation of cortical p-NfM/H and (**c**, **d**) NfL immunoreactivity with cortical thickness on the left, and cortical MD on the right**.** Immunoreactivity of both neurofilaments correlated with decreased cortical thickness, specifically in PD, and with higher cortical MD, specifically in PDD/DLB. Every data point represents a cortical region of interest of a donor, and the groups are indicated by colours in the figure. For each group, the regression line is shown, together with its colour-coded standard error (pink for PD and brown for PDD/DLB). Significant correlations are highlighted in red bold in every panel. Legend: correlation significant at *P < 0.05, **P < 0.01, ***P < 0.001. DLB: Dementia with Lewy bodies; FWC-MD: free-water corrected mean diffusivity; MD: mean diffusivity; NfL: neurofilament light chain; PD: Parkinson’s disease; PDD: Parkinson’s disease dementia; p-NfM/H: phosphorylated neurofilament medium and heavy chain

P-NfM/H immunoreactivity negatively correlated with cortical thickness in the full PD + PDD/DLB cohort across all cortical regions (*r* = − 0.23, *R*^2^ = 5%, *P* = 0.020). Particularly, the association was driven by the PD group (*r* = − 0.32, *R*^2^ = 10%, *P* = 0.047), and not significant in PDD/DLB (*r* = − 0.21, *P* = 0.106) (Fig. 5a). No correlations were found in the control group (*P* > 0.05), or for individual brain regions (*P*-FDR > 0.05).

Additionally, NfL immunoreactivity negatively correlated with cortical thickness in the full PD + PDD/DLB cohort across all cortical regions (*r* = − 0.22, *R*^2^ = 5%, *P* = 0.012). Again, the association was driven by PD (*r* = − 0.27, *R*^2^ = 7%, *P* = 0.025), and absent in PDD/DLB (*r* = − 0.15, *P* = 0.220) (Fig. 5c). No correlations were found in the control group (*P* > 0.05), or for individual brain regions (*P*-FDR > 0.05).

Taken together, cortical thickness was not sensitive to pathological load. However, both p-NfM/H and NfL immunoreactivity levels correlated with cortical thickness, especially in the PD group.

### Cortical neurofilament immunoreactivity is reflected by cortical diffusivity in PDD/DLB

To investigate whether cortical MD is sensitive to regional neurofilament variation in the cortex of PD and PDD/DLB donors, we assessed whether regional neurofilament immunoreactivity correlated with MD across the cortical regions of interest.

Neither PD (*P* = 1.000) nor PDD/DLB donors (*P* = 0.487) showed overall cortical MD differences compared to controls across and within the regions analysed (*P*-FDR > 0.05) (Additional file 2: Fig. S9c and d). Overall, cortical MD positively correlated with pSer129-αSyn (*r* = 0.32, *R*^2^ = 10%, *P* = 0.002) and p-tau pathology (*r* = 0.36, *R*^2^ = 13%, *P* < 0.001) in PDD/DLB across all cortical regions, but not with Aβ load (*P* = 0.339) (Additional file 2: Fig. S10b and d, and Additional file 1: Table S8).

P-NfM/H immunoreactivity did not correlate with cortical MD in the full PD + PDD/DLB cohort across all cortical regions (*P* = 0.182). However, p-NfM/H immunoreactivity positively correlated with cortical MD in the PDD/DLB group (*r* = 0.28, *R*^2^ = 8%, *P* = 0.003) (Fig. 5b). No correlations were found in the control group (*P* = 0.430), or the PD group (*P* = 0.586), or for individual brain regions (*P*-FDR > 0.05).

Additionally, NfL immunoreactivity positively correlated with cortical MD in the full PD + PDD/DLB cohort across all cortical regions (*r* = 0.21, *R*^2^ = 4%, *P* = 0.024). Particularly, the association was present in the PDD/DLB group (*r* = 0.41, *R*^2^ = 17%, *P* = 0.003), but not in the PD (*P* = 0.184) or control group (*P* = 0.738) (Fig. 5d). No correlations were found for individual brain regions (*P*-FDR > 0.05).

Taken together, cortical MD is a sensitive marker for pSer129-αSyn and p-tau load. In addition, both p-NfM/H and NfL immunoreactivity correlated with increased cortical MD, especially in the PDD/DLB group.

## Discussion

Using post-mortem within-subject in-situ MRI and histopathology approaches [34], we investigated cortical regional neurofilament immunoreactivity and its relation to pathological hallmarks and MRI outcome measures in PD and PDD/DLB from a microscale to a mesoscale level. We found that p-NfM/H was increased in PD and PDD/DLB across almost all cortical regions, while NfL was increased specifically in the parahippocampal gyrus and entorhinal cortex of PDD/DLB donors. NfL accumulated in somas of diseased neurons showing neurodegenerative morphologies, and in axons showing fragmentation. Furthermore, we found that in PDD/DLB, p-NfM/H immunoreactivity levels positively correlated with p-tau load, whereas NfL positively correlated with pSer129-αSyn pathology and more strongly with p-tau load. Lastly, we showed that neurofilament immunoreactivity correlated with cortical thinning in PD and with higher cortical MD in PDD/DLB.

**Fig. 6:**
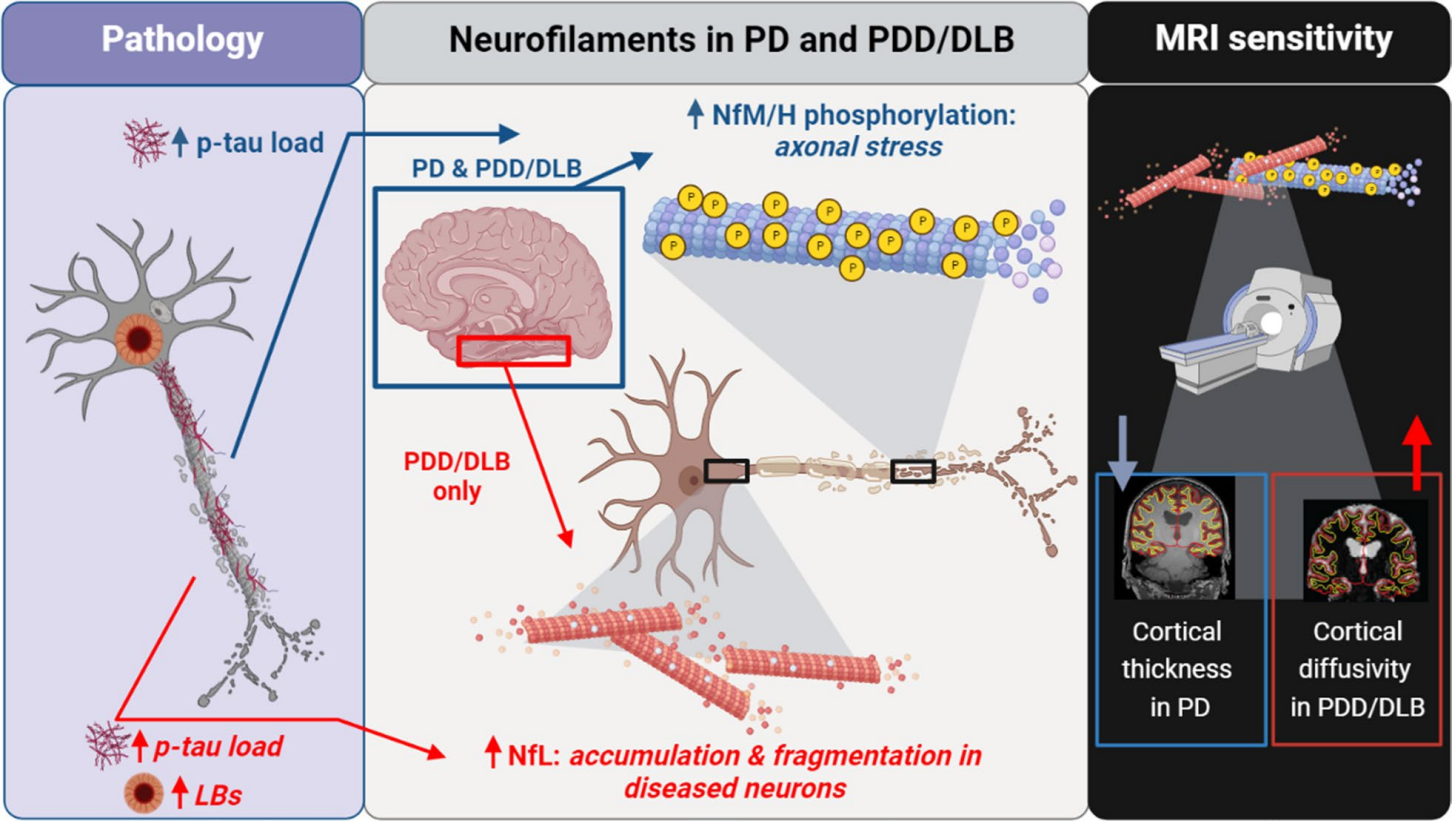
Increased neurofilament immunoreactivity is associated with pathology load and MRI biomarkers of neurodegeneration in PD. Middle panel: neurofilament immunoreactivity is increased in both PD and PDD/DLB cortex. Specifically, increased NfM/H phosphorylation occurs across the cortex in both PD and PDD/DLB (blue square), possibly indicating axonal stress, and is associated with increased p-tau load (left panel, blue arrow). On the other hand, an increased NfL immunoreactivity, representing NfL accumulation in disease neurons and fragmented axons, is observed in the entorhinal cortex and parahippocampal gyrus of PDD/DLB donors (red square) and associated with LB count and p-tau load (left panel, red arrow), suggesting structural changes with increasing pathological burden, and confirming its important role in the context of cognitive decline. Right panel: both increased p-NfM/H (i.e. axonal stress) and NfL immunoreactivity (i.e. NfL accumulation and fragmentation) are reflected by cortical thinning in PD and increased cortical MD in PDD/DLB. Figure created with BioRender.com. DLB: Dementia with Lewy Bodies; LB: Lewy Body; NfL: neurofilament light chain; PD: Parkinson’s disease; PDD: Parkinson’s disease dementia; NfM/H: phosphorylated neurofilament medium and heavy chain; p-tau: phosphorylated tau

For p-NfM/H, we found that both PD and PDD/DLB donors had higher p-NfM/H immunoreactivity levels compared to controls, and that this increase was uniform across the analysed cortical areas. An increase in phosphorylation of neurofilament medium and heavy chains (NfM/H) has been described in several neurological disorders, such as AD, multiple sclerosis, amyotrophic lateral sclerosis and stroke [58], but it has never been investigated in the cerebral cortex of PD patients. Phosphorylation is assumed to be the dominant phosoform of both NfM and NfH, and under normal conditions axons show extensive neurofilament phosphorylation, while there is little or no phosphorylation in the neuronal soma and its dendrites [58, 59], which we also show in our study. Neurofilament phosphorylation is a highly regulated and complex process, and increased phosphorylation of subunits M (medium) and H (heavy) has been linked to several pathological processes [58, 59], even if its function in disease is still under debate. The increase in p-NfM/H immunoreactivity we found may indicate two processes: (i) an increase in the amount of axons or (ii) an increase in phosphorylation of NfM/H. While the first option is unlikely in neurodegenerative diseases, the second has been widely described and suggests that neurofilament hyper-phosphorylation might be linked to axonal transport impairment [58, 59]. Furthermore, a putative neurofilament hyper-phosphorylation might be a by-product of dysregulated kinase activity due to stress factors, such as chronic oxidant stress in neurodegenerative diseases [59]. Cellular stress factors are associated with extensive phosphorylation of cytoskeletal elements through several proline-directed kinases, which are capable of phosphorylating neurofilaments and tau [59, 60], and have been shown to be dysregulated in AD and PD [60, 61]. Moreover, we found that both p-NfM/H and NfL correlated to p-tau load in PDD/DLB, suggestive of cytoskeletal alterations in axons in cortical brain regions [59, 60]. However, more data should be gathered by future research to investigate neurofilament hyper-phosphorylation in PD. Interestingly, we found that p-NfM/H immunoreactivity was higher in males compared to female donors. Males also show a higher prevalence of PD, especially between 50 and 59 years old [62]. Since we show that PD(D) and DLB donors had higher p-NfM/H immunoreactivity, our data suggests that an innate increased p-NfM/H immunoreactivity in males might be related to the higher predominance of PD in this sex group. However, these results need to be taken with caution as the study was not designed to answer this specific research question. Taken together (Fig. 6), our data suggests that the NfM/H subunits might be hyper-phosphorylated across the cortex not only in PDD/DLB, but also in PD donors with long disease duration, suggesting that NfM/H hyper-phosphorylation might be a marker of axonal stress. However, this should be confirmed in future studies.

Furthermore, we found that PDD/DLB cases had higher cortical NfL immunoreactivity levels compared to age-matched controls, and that this increase was specific to the entorhinal cortex and parahippocampal gyrus. An increase in NfL immunoreactivity seems counterintuitive, as it is generally believed that the breakdown of axons leads to an increase of this marker in the extracellular fluid, and therefore a decrease of it in brain tissue [15, 63]. However, higher NfL immunoreactivity has been described before in an animal model and in the brain tissues of stroke patients [64, 65]. Specifically, it has been shown that the same NfL antibody used in this study, the polyclonal rabbit NfL (AA 1–284), not only targets NfL, but most importantly also targets its degradation products [65]. Translating this information to our study, we recorded an increase of NfL immunoreactivity in PDD/DLB, which may result from increased NfL degradation products. In support of this, we also found that NfL accumulated in neuronal somas that showed patterns of neurodegeneration (e.g., nuclear material fragmentation, ballooned and corkscrew cell bodies), and NfL-positive axons often showed fragmentation, as described before in a mouse model of stroke [64, 65]. Taken together, increased NfL in parahippocampal regions seems to point towards evidence of neurofilament accumulation and fragmentation in PDD and DLB. These results are consistent with several CSF and plasma studies, which report that higher CSF or plasma NfL levels correlate with cognitive decline in PD [16–18, 20, 23]. The information that we add to these studies is regional, by showing that NfL immunoreactivity is specifically increased in the entorhinal and parahippocampal cortex, regions that are strongly involved in cognitive processes [66], and are the first to show p-tau accumulation in the adult and aged human brain [53]. This process might therefore hint towards why NfL CSF and plasma levels are increased in several neurological disorders which are per definition p-tau positive, such as AD, frontotemporal dementia and multiple system atrophy [15, 16, 63]. Moreover, besides NfL correlation with p-tau load, we show that NfL immunoreactivity levels were also associated with pSer129-αSyn load. CSF NfL levels have been shown to positively correlate with CSF αSyn [22] and with decline in memory, attentional and executive functioning in PD [24]. Additionally, we recently showed that neurofilaments accumulate and cluster around the core of Lewy bodies in post-mortem brain tissue of PD and PDD/DLB donors [67], illustrating a close relationship between NfL and pathological αSyn, suggesting a role for neurofilament in encapsulating toxic proteins. Taken together (Fig. 6), aggregation and fragmentation of NfL, and its association to p-tau and pSer129-αSyn pathology, suggest that NfL undergoes structural changes with increasing pathological burden, confirming its important role in the context of cognitive decline**.**

To investigate the possibility of measuring the above-mentioned pathological changes in clinical practice, we evaluated whether cortical pathology and neurofilament immunoreactivity would be reflected by MRI biomarkers of neurodegeneration, including cortical thickness and cortical microstructural integrity. We found that increased neurofilament immunoreactivity correlated with lower cortical thickness in PD and with higher cortical MD in PDD/DLB. This discrepant association in non-demented and demented cases may be explained by the fact that we found cortical MD to be a more sensitive marker than cortical thickness in picking up pSer129-αSyn and p-tau pathology, which was more abundantly present in PDD/DLB than in non-demented PD. Moreover, we found an association between neurofilament immunoreactivity and reduced cortical thickness in PD patients, in the absence of obvious cortical atrophy. This was to be expected, since cortical degeneration on MRI has been shown to be extensive but very subtle in PD [30], and only MRI studies with large sample sizes would be able to pick up such changes.

Our results partially go in line with previous literature, where serum NfL was shown to correlate with cortical thinning of several brain areas in *de-novo* PD, but also with increased cortical MD [33]. Unfortunately, no similar studies have been carried out in PDD and DLB, making this the first study to investigate this relationship. Taken together (Fig. 6), we found that cortical thinning in PD, and lower microstructural integrity in PDD/DLB seem to reflect not only accumulation of neurofilaments (i.e., increased NfL), but also axonal stress (i.e., increased p-NfH/M).

The main strength of this study is the combination of MRI and gold-standard histological data of multiple cortical regions, which were collected from well-characterized donors. All donors had pathological confirmation of clinical diagnosis, as clinical-pathological discrepancies occur in about 15% of cases and might obscure *in-vivo* studies [68, 69]. Moreover, we both quantitatively and qualitatively studied regional cortical neurofilament changes in PD. NfL is widely used as a CSF or blood biomarker to detect axonal degeneration in patient cohorts, and with this study we hope to shed light on the regional and morphological changes in neurofilaments, linking neurodegenerative features from micro- to mesoscale and to the clinic. Therefore, results of this study provide evidence for the important roles of neurofilaments in neurodegeneration and their MRI signatures.

There are also some limitations. Neurofilament changes are not a process specific to PD(D) or DLB. In fact, increased CSF and plasma NfL levels have been described in several other neurological disorders compared to age-matched controls, and are considered a measure of neuroaxonal damage independent of casual pathways [15, 16]. In addition, this study focused on neurofilament changes of only 7 cortical regions and their correlations to cortical thickness, rather than subcortical neurofilament changes and their correlations with volume, even if differential neurofilament immunoreactivity might be found at this level. In fact, PD patients might show higher NfL accumulation in the substantia nigra rather than the cortex, and DLB patients might have differential NfL accumulation in the basal ganglia and the occipital cortex. Moreover, neurofilament changes only account for part of the variance of the damage seen on MRI and DTI (in our study up to 17%), and it is likely that other cellular and molecular components contribute to neurodegeneration, such as synaptic loss or glial changes. In addition, we did not find associations between MRI outcome measures and neurofilament immunoreactivity within the included brain regions (only across). This lack of regional sensitivity to neurofilament changes might be due to our small sample size, since large PD(D) cohorts are needed to pick up subtle changes in cortical thinning [30]. Of note, even though post-mortem diffusivity measures were acquired in situ (brain in cranium), which is closer to the in vivo brain scanning than the ex vivo setting (brain extracted from the cranium), several factors (i.e., lower body temperature, tissue decomposition and partial volume effects of decreased CSF diffusivity, swelling, hypoxia) may influence the diffusivity properties [55, 56]. Therefore, even though we took into account post-mortem delay as a covariate in our analyses, this should be kept in mind in the comparisons between post-mortem and in vivo studies. Taken together, future research should therefore be performed to investigate the mechanisms behind the increase in neurofilaments and their association with MRI biomarkers in larger cohorts and in other neurological disorders showing high NfL levels and more pronounced regional atrophy, such as AD, frontotemporal dementia and multiple system atrophy [15, 16].

## Conclusions

Taken together, we provide evidence for increased phosphorylation of NfM/H, suggesting axonal stress across the cortex of PD, PDD and DLB donors. Furthermore, we show that NfL immunoreactivity is increased in the parahippocampal gyrus and entorhinal cortex of PDD and DLB donors, reflecting aggregation and fragmentation of NfL in diseased neurons and axons, suggesting that NfL undergoes structural changes with increasing pathological burden in the context of cognitive decline. Importantly, we provide evidence for the relation between pathological burden and neurofilament levels, and demonstrate that such microscopic markers at least partly explain MRI markers that are associated with the neurodegenerative process.

## Supplementary Information

**Additional file 1**. **Supplementary tables. Table S1.** Donor characteristics. **Table S2.** Concatenated labels from Lausanne atlas for the regions of interest of this study. **Table S3. **Information on primary and secondary antibodies. **Table S4. **Correlations between pathological hallmarks. **Table S5. **Correlations of p-NfM/H immunoreactivity with pathology load in superficial (layer I-III) and deep cortical layers (layer IV-VI). **Table S6. **Correlations of NfL immunoreactivity with pathology load in superficial (layer I-III) and deep cortical layers (layer IV-VI). **Table S7.** Correlations of MRI-derived cortical thickness with neurofilaments immunoreactivity and pathology load. **Table S8.** Correlations of DTI-derived cortical MD with neurofilaments immunoreactivity and pathology load.

**Additional file 2**. **Supplementary figures.** **Fig. S1.** Segmentation of entorhinal cortex, parahippocampal and fusiform gyrus in hippocampal section. **Fig. S2.** Quantification of pathology load using QuPath algorithms. **Fig. S3.** Photographs of representative sections of controls, PD and PDD/DLB donors stained for NfL and p-NfM/H. **Fig. S4.** Cortical pathology load in controls, PD and PDD/DLB donors. **Fig. S5.** Differences in cortical neurofilaments immunoreactivity in sex, and neurofilaments association with age. **Fig. S6.** Cortical neurofilaments immunoreactivity in superficial (layer I-III) and deep cortical layers (layer IV-VI). **Fig. S7.** Cortical neurofilaments correlation in superficial (layer I-III) and deep cortical layers (layer IV-VI). **Fig. S8.** Neurofilaments immunoreactivity in the parahippocampal gyrus correlates with pathological staging. **Fig. S9.** Cortical thickness and MD do not differ between controls, PD and PDD/DLB donors. **Fig. S10.** Cortical MD, but not cortical thickness, correlates with pSer129-αSyn and p-tau pathology.

**Additional file 3**. **Supplementary materials.** Pathological pSer129-αSyn quantification.

## Data Availability

The data that support the findings of this study are available from the corresponding author, upon reasonable request.

## Abbreviations

Aβ: Amyloid-beta
AD: Alzheimer’s disease
CSF: Cerebrospinal fluid
DLB: Dementia with Lewy bodies
DTI: Diffusion tensor imaging
FDR: False discovery rate
FWC-MD: Free-water corrected mean diffusivity
IHC: Immunohistochemistry
LB: Lewy body
MD: Mean diffusivity
NfL: Neurofilament light chain
NFT: Neurofibrillary tangles
PD: Parkinson’s disease
PDD: Parkinson’s disease dementia
p-NfM/H: Phosphorylated neurofilament medium and heavy chain
p-tau: Phosphorylated-tau
pSer129-αSyn: Phosphorylated Ser129 alpha synuclein

## Declarations

### Declarations

**Ethical approval and consent to participate.**

All donors signed an informed consent for brain donation and the use of material and clinical information for research purposes. The procedures for brain tissue collection of NBB and NABCA have been approved by the Medical Ethical Committee of Amsterdam UMC, Vrije Universiteit Amsterdam.
