# Supplementary material for: Neurofilament light chain is increased in the parahippocampal cortex and associates with pathological hallmarks in Parkinson’s disease dementia
Source: Transl Neurodegener. 2023 Jan 20;12:3. doi: 10.1186/s40035-022-00328-8 (PMC9854202; doi:10.1186/s40035-022-00328-8)
Supplement: Supplementary file 1 — Additional file 1. Supplementary tables. Table S1. Donor characteristics. Table S2. Concatenated labels from Lausanne atlas for the regions of interest of this study. Table S3. Information on primary and secondary antibodies. Table S4. Correlations between pathological hallmarks. Table S5. Correlations of p-NfM/H immunoreactivity with pathology load in superficial (layer I-III) and deep cortical layers (layer IV-VI). Table S6. Correlations of NfL immunoreactivity with pathology load in superficial (layer I-III) and deep cortical layers (layer IV-VI). Table S7. Correlations of MRI-derived cortical thickness with neurofilaments immunoreactivity and pathology load. Table S8. Correlations of DTI-derived cortical MD with neurofilaments immunoreactivity and pathology load. [file 40035_2022_328_MOESM1_ESM.pdf]

Supplementary tables

Table S1. Donor characteristics.

Case number	Clinical diagnosis	Sex	Age at death (years)	Disease duration (years)	PMD (hr:min)	Cause of death	3DT1 scan	DTI scan	IHC	NBV (L)	NGMV (L)	ABC score [5]	Thal phase [6]	Braak NFT stage [1]	Braak LB stage [2]
**Controls**															
1	Ctrl	M	68	-	8:30	Euthanasia	x		x	1,62	0,86	A1B1C0	2	1	0
2	Ctrl	F	63	-	8:10	Euthanasia	x	x	x	1,53	0,75	A0B0C0	0	0	0
3	Ctrl	M	82	-	10:40	Liver cirrhosis	x	x	x	1,40	0,68	A1B1C0	1	1	0
4	Ctrl	M	85	-	9:22	Euthanasia	x	x	x	1,36	0,68	A1B1C0	1	1	0
5	Ctrl	M	67	-	7:35	Euthanasia	x	x	x	1,51	0,74	A1B1C0	1	2	0
6	Ctrl	F	76	-	7:50	Euthanasia	x	x	x	1,47	0,78	A1B1C0	2	1	0
7	Ctrl	M	67	-	8:10	Liver cirrhosis	x	x	x	1,45	0,74	A1B1C0	1	1	0
8	Ctrl	F	77	-	9:00	Euthanasia	x	x	x	1,41	0,74	A1B1C0	1	2	0
9	Ctrl	F	87	-	8:35	Urinary tract infection	x		x	1,37	0,65	A0B1C0	0	1	0
10	Ctrl	F	72	-	7:20	Heart failure	x		x	1,52	0,80	A0B0C0	0	0	0
11	Ctrl	F	69	-	12:45	Pulmonary embolism	x		x	1,39	0,71	A1B1C0	1	1	1
12	Ctrl	M	59	-	8:00	Euthanasia	x		x	1,49	0,76	A1B1C0	2	1	0
13	Ctrl	M	77	-	11:25	Pneumonia	x	x	x	1,49	0,72	A1B1C0	1	1	0
14	Ctrl	F	78	-	10:00	Unknown	x	x	x	1,51	0,77	A1B1C0	1	1	1
15	Ctrl	F	77	-	4:35	Unknown	x	x	x	1,46	0,76	A1B1C0	2	2	2
16	Ctrl	F	59	-	8:10	Euthanasia	x	x	x	1,46	0,78	A0B0C0	0	0	0
17	Ctrl	F	71	-	6:50	Lung carcinoma	x	x	x	1,47	0,76	A1B1C0	2	1	0
18	Ctrl	M	74	-	10:20	Euthanasia	x	x	x	1,39	0,70	A2B1C0	3	2	0
**PD**															
1	PD	F	83	22	10:35	Euthanasia	x	x	x	1,42	0,68	A0B1C0	0	1	5

2	PD	F	69	15	07:05	Aspiration pneumonia	x	x	x	1,51	0,73	A1B1C0	2	2	6
3	PD	F	82	17	09:17	Aspiration pneumonia	x	x	x	1,44	0,73	A1B1C0	2	2	6
4	PD	M	78	17	07:15	Euthanasia	x	x	x	1,45	0,76	A1B1C0	1	2	6
5	PD	M	92	16	10:10	Myocardial infarction	x	x	x	1,38	0,70	A2B2C1	3	3	4
6	PD	M	75	20	4:55	End-stage PD	x	x	x	1,41	0,74	A2B1C0	3	2	6
7	PD	M	78	20	3:30	End-stage PD	x	x	x	1,25	0,63	A1B1C0	1	2	6
8	PD	M	70	8	6:55	Euthanasia	x	x	x	1,53	0,78	A1B1C0	1	1	6
9	PD	M	93	23	10:40	Euthanasia	x	x	x	1,50	0,72	A1B2C0	2	4	6
**PDD/ DLB**															
1	PDD	F	83	16	10:40	End-stage PDD	x	x	x	1,33	0,65	A3B2C2	4	4	6
2	DLB	M	66	4	08:00	Euthanasia	x	x	x	1,49	0,73	A3B2C2	4	4	6
3	PDD	F	94	10	06:50	Femur fracture	x	x	x	1,33	0,68	A3B2C2	4	4	6
4	PDD	F	74	12	08:10	Euthanasia	x	x	x	1,33	0,71	A2B1C0	3	2	6
5	DLB	M	72	5	07:10	Euthanasia	x	x	x	1,42	0,72	A2B1C2	3	2	6
6	DLB	M	77	7	07:15	End-stage DLB	x	x	x	1,45	0,75	A1B2C0	1	3	6
7	PDD	M	79	21	09:25	Subarachnoid bleeding	x	x	x	1,38	0,71	A2B1C1	3	2	6
8	PDD	M	62	18	05:10	End-stage PDD	x		x	1,37	0,71	A1B1C0	1	2	6
9	PDD	M	74	8	8:50	Aspiration pneumonia	x	x	x	1,29	0,62	A1B2C0	2	3	6
10	PDD	F	81	19	5:30	End-stage PDD	x	x	x	1,35	0,66	A2B1C0	3	2	6
11	DLB	M	91	3	4:30	Euthanasia	x	x	x	1,48	0,72	A2B2C2	3	4	6
12	DLB	F	86	6	7:00	Dehydration, cachexia	x	x	x	1,31	0,69	A3B2C2	5	4	6

Legend: *3DT1: post-mortem in-situ MRI 3DT1 weighted scan; Ctrl: control; DLB: Dementia with Lewy Bodies; DTI: diffusion tensor imaging; F: female; hr: hour; IHC: immunohistochemistry; L: liter; LB: Lewy Body; M: male; min: minute; NBV: normalized brain volume; NFT: neurofibrillary tangles; NGMV: normalized grey matter volume; PD: Parkinson's disease; PDD: Parkinson's disease dementia; PMD: post-mortem delay.*

Table S2. Concatenated labels from Lausanne atlas for the regions of interest of this study.

Region of interest	Labels Lausanne atlas [3, 4]
Superior frontal	Superiorfrontal_4 + Superiorfrontal_5 + Superiorfrontal_6
Anterior cingulate	Caudalanteriorcingulate_2 + Caudalanteriorcingulate_3
Posterior cingulate	Posteriorcingulate_3 + Posteriorcingulate_4
Superior parietal	Superiorparietal_1 + Superiorparietal_2 + Superiorparietal_3
Parahippocampal	Parahippocampal_3
Entorhinal	Entorhinal_1
Fusiform	Fusiform_7
Middle temporal	Middletemporal_5 + Middletemporal_7
Anterior insula	Insula_3 + Insula_4

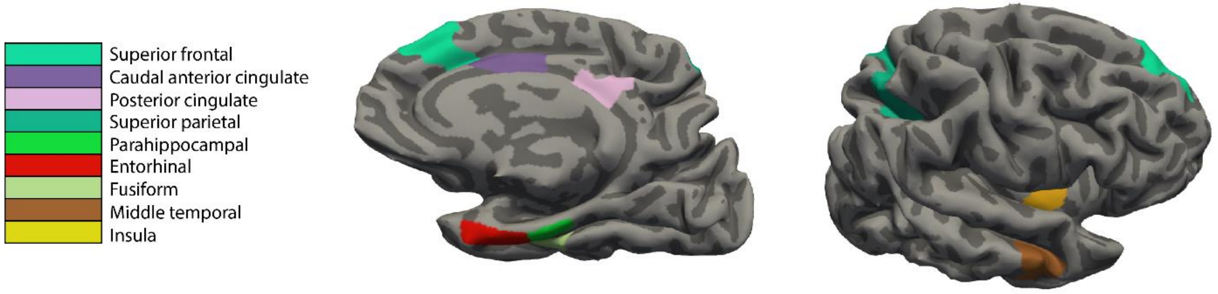

Table S3. Information on primary and secondary antibodies.

Antibody	**Antigen**	**Species**	**Origin details**	**Dilution**	**Incubation time**	**Antigen retrieval**	**Detection method**
Primary antibodies							
pSer129 aSyn, clone EP1536Y	Alpha synuclein phosphorylated at Ser129	Rabbit igG	Abcam, Cambridge, UK	1:8000 for BF<br>1:4000 for FL	4°C o.n.	Tris EDTA buffer (pH 9.0) in steam cooker	EnVision (HRP)
Aβ, clone 4G8	Aβ amino acid sequence 17-24	Mouse igG2b	BioLegend, San Diego, USA	1:8000	4°C o.n.	Citrate buffer (pH 6.0) in steam cooker	EnVision (HRP)
p-tau, clone AT8	Tau phosphorylated at Ser202 and Thr205	Mouse igG1	ThermoFisher, Pittsburgh, USA	1:800 for BF<br>1:800 for FL	4°C o.n.	Citrate buffer (pH 6.0) in steam cooker	EnVision (HRP)
NfL	Immunogen corresponds to AA 1 to 284 (with AA 200-292 missing)	Rabbit igG	Synaptic systems, Göttingen, Germany	1:600 for BF<br>1:200 for FL	4°C o.n.	Tris EDTA buffer (pH 9.0) in steam cooker	EnVision (HRP)
SMI312	Mixture of monoclonal antibodies directed against phosphorylated NfM and NfH.	Mouse igG1	BioLegend, San Diego, USA	1:500 for BF<br>1:500 for FL	4°C o.n.	Tris EDTA buffer (pH 9.0) in steam cooker	EnVision (HRP)
Secondary antibodies	**Host species**	**Target species**	**Origin details**	**Dilution**	**Incubation time**	**Antigen retrieval**	**Detection method**
DoAM Alexa 488	Donkey IgG	Mouse	ThermoFisher, Pittsburgh, USA	1:200	2 hrs at RT	/	fluorochrome
DoAR Alexa 594	Donkey IgG	Rabbit	ThermoFisher, Pittsburgh, USA	1:200	2 hrs at RT	/	fluorochrome

Legend: *BF: bright-field; FL: fluorescence; hrs: hours; o.n.: overnight; RT: room temperature.*

Table S4. Correlations between pathological hallmarks.

	Controls	PD + PDD/DLB	PD	PDD/DLB
LB count (LB/mm²)				
p-αSyn load (%)	-	**p<0.001, r = 0.89**	**p<0.001, r = 0.84**	**p<0.001, r = 0.88**
p-tau load (%)	-	**p<0.001, r = 0.63**	p=0.611, r = 0.06	**p<0.001, r = 0.68**
Aβ load (%)		p=0.909, r = -0.01	p=0.282, r = -0.13	p=0.999, r = 0.00
pSer129-αSyn load (%)				
p-tau load (%)	-	**p<0.001, r = 0.57**	p=0.607, r = 0.07	**p<0.001, r = 0.60**
Aβ load (%)	-	p=0.365, r = -0.05	p=0.670, r = -0.05	p=0.369, r = -0.06
p-tau load (%)				
Aβ load (%)	p=0.280, r = -0.01	p=0.687, r = 0.02	p=0.623, r = 0.05	p=0.906, r = 0.01

For each correlation, p-value and correlation coefficients are shown. When the correlation is significant it is highlighted in bold. **Legend**: *Aβ: amyloid beta; DLB: Dementia with Lewy Bodies; LB: Lewy Body; PD: Parkinson's disease; PDD: Parkinson's disease dementia; pSer129-αSyn: phosphorylated Ser129 alpha synuclein; p-tau: phosphorylated tau.*

Table S5. Correlations of p-NfM/H immunoreactivity with pathology load in superficial (layer I-III) and deep cortical layers (layer IV-VI).

	**Controls**	**PD + PDD/DLB**	**PD**	**PDD/DLB**
p-NfM/H immunoreactivity (%)				
LB count (LB/mm²) : entire cortex	p=0.187, r = 0.15	p=0.525, r = 0.07	p=0.319, r = 0.15	p=0.920, r = 0.01
superficial	p=0.650, r = 0.04	p=0.416, r = 0.08	p=0.711, r = 0.06	p=0.444, r = 0.11
deep	p=0.214, r = -0.13	p=0.567, r = 0.05	p=0.132, r = 0.19	p=0.907, r = -0.01
pSer129-αSyn load (%): entire cortex	p=0.760, r = 0.03	p=0.807, r = 0.03	p=0.381, r = 0.13	p=0.825, r = -0.03
superficial	p=0.904, r = 0.01	p=0.834, r = 0.02	p=0.823, r = 0.03	p=0.856, r = -0.02
deep	p=0.370, r = 0.09	p=0.886, r = 0.01	*p=0.088, r = 0.22*	p=0.728, r = -0.04
p-tau load (%): entire cortex	p=0.149, r = -0.18	**p=0.018, r = 0.26**	*p=0.066, r = 0.32*	**p=0.043, r = 0.29**
superficial	p=0.707, r = -0.06	p=0.704, r = 0.05	p=0.168, r = 0.34	p=0.608, r = -0.09
deep	p=0.581, r = -0.11	p=0.361, r = -0.13	p=0.758, r = 0.06	p=0.283, r = -0.22
Aβ load (%): entire cortex	p=0.737, r = -0.03	p=0.441, r = -0.06	p=0.725, r = 0.05	p=0.248, r = -0.12
superficial	p=0.603, r = -0.06	p=0.196, r = -0.10	p=0.776, r = -0.04	p=0.096, r = -0.18
deep	p=0.892, r = 0.02	p=0.784, r = -0.02	p=0.683, r = -0.07	p=0.964, r = 0.01

For each correlation, p-value and correlation coefficients are shown. When the correlation is significant it is highlighted in bold. Correlations coloured in italics are trends. For each marker, the correlations in the entire cortex (layers I-VI), superficial (I-III) and deep (IV-VI) cortical layers are shown. **Legend**: *Aβ: amyloid beta; DLB: Dementia with Lewy Bodies; LB: Lewy Body; PD: Parkinson's disease; PDD: Parkinson's disease dementia; pSer129-αSyn: phosphorylated Ser129 alpha synuclein; p-NfM/H: phosphorylated neurofilament medium and heavy chain; p-tau: phosphorylated tau.*

Table S6. Correlations of NfL immunoreactivity with pathology load in superficial (layer I-III) and deep cortical layers (layer IV-VI).

	**Controls**	**PD + PDD/DLB**	**PD**	**PDD/DLB**
NfL immunoreactivity (%)				
LB count (LB/mm²): entire cortex	p=0.658, r = 0.05	**p<0.001, r = 0.36**	p=0.392, r = 0.10	**p=0.002, r = 0.40**
superficial	p=0.851, r = 0.02	**p<0.001, r = 0.28**	p=0.649, r = 0.05	**p=0.005, r = 0.33**
deep	p=0.144, r = 0.14	**p=0.005, r = 0.24**	p=0.805, r = 0.02	**p=0.025, r = 0.27**
pSer129-αSyn load (%): entire cortex	p=0.666, r = -0.04	**p<0.001, r = 0.30**	p=0.910, r = 0.01	**p=0.005, r = 0.36**
superficial	p=0.881, r = 0.01	**p=0.006, r = 0.22**	p=0.997, r = 0.00	**p=0.021, r = 0.26**
deep	p=0.680, r = -0.04	**p=0.046, r = 0.16**	p=0.217, r = -0.12	*p=0.074, r = 0.21*
p-tau load (%): entire cortex	p=0.579, r = -0.06	**p<0.001, r = 0.42**	**p=0.014, r = 0.31**	**p<0.001, r = 0.42**
superficial	p=0.765, r = 0.04	p=0.112, r = 0.18	p=0.872, r = 0.02	p=0.329, r = 0.15
deep	p=0.828, r = 0.03	p=0.436, r = 0.08	p=0.802, r = -0.09	p=0.866, r = 0.03
Aβ load (%): entire cortex	p=0.831, r = 0.02	p=0.499, r = -0.43	p=0.325, r = -0.09	p=0.652, r = -0.04
superficial	p=0.461, r = -0.07	*p=0.051, r = 0.11*	p=0.145, r = -0.10	*p=0.063, r = 0.15*
deep	**p=0.034, r = -0.27**	p=0.924, r = 0.01	p=0.374, r = 0.11	p=0.819, r = -0.02

For each correlation, p-value and correlation coefficients are shown. When the correlation is significant, it is highlighted in bold, and the strength of the correlation is given by a green shade colour (dark green moderate correlation, light green is a weak correlation). Correlations in italics are trends. For each marker, the correlations in the entire cortex (layers I-VI), superficial (I-III) and deep (IV-VI) cortical layers are shown. **Legend**: *Aβ: amyloid beta; DLB: Dementia with Lewy Bodies; LB: Lewy Body; NfL: neurofilament light chain; PD: Parkinson's disease; PDD: Parkinson's disease dementia; pSer129-αSyn: phosphorylated Ser129 alpha synuclein; p-tau: phosphorylated tau.*

Table S7. Correlations of MRI-derived cortical thickness with neurofilaments immunoreactivity and pathology load.

	Controls	PD + PDD/DLB	PD	PDD/DLB
Cortical thickness (Z score)				
p-NfM/H immunoreactivity (%)	p=0.686, r = 0.04	**p=0.020, r = -0.23**	**p=0.047, r = -0.32**	p=0.106, r = -0.21
NfL immunoreactivity (%)	p=0.680, r = 0.33	**p=0.012, r = -0.22**	**p=0.025, r = -0.27**	p=0.220, r = -0.15
LB count (LB/mm^2)	-	p=0.910, r = -0.01	p=0.506, r = -0.08	p=0.615, r = -0.05
pSer129-αSyn load (%)	-	p=0.784, r = 0.02	p=0.781, r = -0.04	p=0.405, r = 0.09
p-tau load (%)	p=0.400, r = 0.06	p=0.106, r = -0.13	*p=0.091, r = -0.20*	p=0.440, r = -0.08
Aβ load (%)	p=0.488, r = 0.68	p=0.876, r = -0.01	p=0.632, r = -0.06	p=0.367, r = 0.12

For each correlation, p-value and correlation coefficients are shown. When the correlation is significant it is highlighted in bold, and the strength of the correlation is given by a green shade colour (dark green moderate correlation, light green is a weak correlation). Correlations in italics are trends. **Legend:** *Aβ: amyloid beta; DLB: Dementia with Lewy Bodies; LB: Lewy Body; PD: Parkinson's disease; PDD: Parkinson's disease dementia; pSer129-αSyn: phosphorylated Ser129 alpha synuclein; p-NfM/H: phosphorylated neurofilament medium and heavy chain; p-tau: phosphorylated tau.*

Table S8. Correlations of DTI-derived cortical MD with neurofilaments immunoreactivity and pathology load.

	Controls	PD + PDD/DLB	PD	PDD/DLB
FWC-MD (10^{-3} mm^2/s)				
p-NfM/H immunoreactivity (%)	p=0.430, r = -0.08	p=0.182, r = 0.14	p=0.586, r = 0.10	**p=0.003, r = 0.28**
NfL immunoreactivity (%)	p=0.738, r = 0.03	**p=0.024, r = 0.21**	p=0.184, r = -0.19	**p=0.003, r = 0.41**
LB count (LB/mm^2)	-	**p=0.002, r = 0.23**	p=0.533, r = -0.07	**p=0.002, r = 0.32**
pSer129-αSyn load (%)	-	**p=0.003, r = 0.23**	p=0.874, r = 0.02	**p=0.002, r = 0.32**
p-tau load (%)	**p=0.003, r = 0.23**	**p<0.001, r = 0.28**	p=0.393, r = 0.10	**p<0.001, r = 0.36**
Aβ load (%)	p=0.779, r = -0.01	p=0.850, r = 0.02	p=0.366, r = -0.11	p=0.339, r = 0.00

For each correlation, p-value and correlation coefficients are shown. When the correlation is significant it is highlighted in bold, and the strength of the correlation is given by a green shade colour (dark green moderate correlation, light green is a weak correlation). **Legend**: *Aβ: amyloid beta; DLB: Dementia with Lewy Bodies; DTI: diffusion tensor imaging; FWC-MD: free-water mean diffusivity; LB: Lewy Body; PD: Parkinson's disease; PDD: Parkinson's disease dementia; pSer129-αSyn: phosphorylated Ser129 alpha synuclein; p-NfM/H: phosphorylated neurofilament medium and heavy chain; p-tau: phosphorylated tau.*

References supplementary tables

1 Braak H, Alafuzoff I, Arzberger T, Kretzschmar H, Del Tredici K (2006) Staging of Alzheimer disease-associated neurofibrillary pathology using paraffin sections and immunocytochemistry. Acta neuropathologica 112: 389-404

2 Braak H, Del Tredici K, Rub U, de Vos RA, Jansen Steur EN, Braak E (2003) Staging of brain pathology related to sporadic Parkinson's disease. Neurobiol Aging 24: 197-211 Doi 10.1016/s0197-4580(02)00065-9

3 Daducci A, Gerhard S, Griffa A, Lemkaddem A, Cammoun L, Gigandet X, Meuli R, Hagmann P, Thiran JP (2012) The connectome mapper: an open-source processing pipeline to map connectomes with MRI. PLoS One 7: e48121 Doi 10.1371/journal.pone.0048121

4 Hagmann P, Cammoun L, Gigandet X, Meuli R, Honey CJ, Wedeen VJ, Sporns O (2008) Mapping the structural core of human cerebral cortex. PLoS Biol 6: e159 Doi 10.1371/journal.pbio.0060159

5 Montine TJ, Phelps CH, Beach TG, Bigio EH, Cairns NJ, Dickson DW, Duyckaerts C, Frosch MP, Masliah E, Mirra SS (2012) National Institute on Aging–Alzheimer's Association guidelines for the neuropathologic assessment of Alzheimer's disease: a practical approach. Acta neuropathologica 123: 1-11

6 Thal DR, Rüb U, Orantes M, Braak H (2002) Phases of Aβ-deposition in the human brain and its relevance for the development of AD. Neurology 58: 1791-1800
